# The role of prenatal maternal sex steroid hormones in weight and adiposity at birth and growth trajectories during infancy

**DOI:** 10.21203/rs.3.rs-4178000/v1

**Published:** 2024-04-10

**Authors:** Ying Meng, Loralei Thornburg, Caitlin Dreisbach, Charlotte Orzolek, Amber Kautz, Hannah Murphy, Zorimar Rivera- Núñez, Christina Wang, Richard Miller, Thomas O’Connor, Emily Barrett

**Affiliations:** University of Rochester; University of Rochester Medical Center; University of Rochester Medical Center; University of Rochester Medical Center; Vizient Inc.; Rutgers University; The Lundquist Institute at Harbor-UCLA Medical Center; University of Rochester Medical Center; University of Rochester; Rutgers University

**Keywords:** testosterone, estrogen, weight trajectory, adiposity trajectory, infants

## Abstract

**Objective:**

Intrauterine factors can impact fetal and child growth and may underlie the developmental origins of childhood obesity. Sex steroid hormone exposure during pregnancy is a plausible target because of the impact on placental vascularization, nutrient transportation, bone growth, adipogenesis, and epigenetic modifications. In this study we assessed maternal sex steroid hormones in each trimester in relation to birthweight, neonatal adiposity, and infant growth trajectories, and evaluate sensitive windows of development.

**Methods:**

Participants from a prospective pregnancy cohort who delivered at term were included in the analysis (n=252). Estrone, estradiol, and estriol, as well as total and free testosterone throughout gestation were assessed using high-performance liquid chromatography and tandem mass spectrometry. Path analyses were used to assess the direct associations of sex steroid hormones in each trimester with birth outcomes and infant growth trajectories (birth to 12 months) adjusting for covariates and considering moderation by sex.

**Results:**

The associations between prenatal sex steroid hormones and fetal/infant growth varied by sex and hormone assessment timing. First trimester estrone were associated with higher birthweight z-scores (β=0.37, 95%CI: 0.02, 0.73) and truncal skinfold thickness (TST) at birth (β=0.94, 95%CI: 0.34, 1.54) in female infants. Third trimester total testosterone was associated with higher TST at birth (β=0.61, 95%CI: 0.02, 1.21) in male infants. First trimester estrone/estradiol and first and third trimesters testosterone were associated with lower probabilities of high stable weight trajectory compared to low stable weight trajectory (Estrone: β=−3.87, 95%CI: −6.59, −1.16; First trimester testosterone: β=−3.53, 95%CI: −6.63, −0.43; Third trimester testosterone: β=−3.67, 95%CI: −6.66, −0.69) during infancy in male infants.

**Conclusions:**

We observed associations between prenatal sex steroid hormone exposure and birthweight, neonatal adiposity and infant growth that were sex and gestational timing dependent. Our findings suggest further investigation on additional mechanisms linking prenatal sex steroid exposure and fetal/postnatal growth is needed.

## Introduction

Childhood obesity has reached epidemic proportions, affecting nearly one in five youths in the United States (1). The risk of childhood obesity is evident in early infancy, as both high and low birthweight as well as rapid weight gain during infancy have been consistently linked to childhood obesity (2–4). Emerging evidence has suggested that prenatal factors, including gestational weight gain, gestational diabetes, and smoking during pregnancy play a role in the development of childhood obesity (5, 6). The underlying mechanism may involve fetal programming, whereby physiological and metabolic set points are modulated by the intrauterine milieu, affecting fetal growth and obesity susceptibility (7). Therefore, identification of intrauterine factors linked to birthweight and infant growth trajectories is critical to understand the early developmental origins of childhood obesity.

Sex steroid hormones are potential key intrauterine factors that modify fetal growth and obesity susceptibility. Sex steroid hormones regulate a wide range of maternal and fetal functions (8, 9), including placental vascularization, endometrial and placental nutrient transporter expression, and epigenetic modification of fetal tissues (9–12). Additionally, estrogens and testosterone can induce cell proliferation, particularly in bone and adipocytes, and thus potentially contribute to offspring growth and adiposity in utero as well as postnatally (13–15). Therefore, early life exposure to sex steroid hormones may contribute to the intrauterine origins of childhood obesity.

The majority of studies assessing sex steroids and birthweight have focused on estriol (E3) due to its widespread availability as part of prenatal screening test, reporting positive associations (16–18). However, fewer studies have examined the relationship between other sex steroid hormones and birthweight and results have been controversial (19–25). Also, the majority of previous studies assessed prenatal sex steroid hormones at a single time point, most often mid-late pregnancy (16, 18, 19, 25). Recognizing that variations in fetal growth originates in early pregnancy, repeated assessment of sex steroids across pregnancy may be suitable to identify critical periods during which fetal growth is most sensitive to endocrine activity. Whether associations persist beyond birth into infancy remains an open question as well (16, 25, 26).

In this study, we leveraged clinical data and biospecimens from a pregnancy cohort to assess maternal sex steroid concentrations (estrone, estradiol, E3, total testosterone, and free testosterone) in each trimester in relation to birthweight, neonatal adiposity, and infant weight and adiposity growth trajectories through the age of 12 months. Additionally, we evaluated moderation by infant sex in light of prior evidence indicating sex differences in fetal growth, placental function and adipose tissue biology (27, 28).

## Methods

### Study Overview

This study analyzed data from a prospective pregnancy cohort, the Understanding Pregnancy Signals and Infant Development (UPSIDE) study, which is part of the Environmental Influences on Child Health Outcomes (ECHO) program (29). The UPSIDE study recruited pregnant people (n = 326) in their first trimester from 2015 to 2019 through University of Rochester Medical Center (Rochester, NY, USA) affiliated obstetric clinics. Eligibility criteria for the current study included: (1) available serum sex steroid hormone data in all trimesters, (2) infants born at term (gestational age ≥ 37 weeks) to assess associations in low-risk population, (3) available data on birth and/or infant growth outcomes. There were 252 mother-child dyads included in the final analyses (Supplementary Fig. 1). The UPSIDE study was approved by the institutional review boards at the University of Rochester and Rutgers University. Written informed consent was obtained from all participants.

### Sex Steroid Hormone Assays

Blood was collected during study visits in each trimester (1st trimester: 12.2 ± 1.3 weeks; 2nd trimester: 21.2 ± 1.8 weeks; 3rd trimester: 31.4 ± 2.0 weeks). The detail of sex steroid assessment was described in our previous study (30). Briefly, sex steroid hormones from serum samples were quantified using validated liquid chromatography with tandem mass spectrometry (LC-MS/MS) methods at the Endocrine and Metabolic Research Laboratory at the Lundquist Institute at Harbor-UCLA Medical Center. Total testosterone (TT) was measured using a Shimadzu HPLC system (Columbia, MD) and an Applied Biosystems API5500 LC–MS/MS (Foster City, CA) equipped with a Turbo-Ion-Spray source with positive mode. Equilibrium dialysis using labeled testosterone was used to quantify free testosterone (fT). The Shimadzu HPLC system (Columbia, MD) and a triple quadrupole mass spectrometer (API5000 LC–MS/MS, Foster City, CA) were used to measure estrogens. The limit of quantification (LOQ) was 50 pg/mL for E3. LOQ/$\sqrt{2}$. was used to replace E3 values less than LOQ/$\sqrt{2}$ or missing (n = 54) in the 1st trimester.

### Birth and Infant Growth Outcomes

We considered four outcomes: (1) birthweight, (2) newborn truncal skinfold thickness (TST [as a surrogate of adiposity]), (3) growth trajectories for weight-for-age percentile, and (4) TST trajectories from birth to 12 months of age. Birthweights were abstracted from medical records and converted to standardized (Z) scores adjusting for gestational age and infant sex according to Fenton growth standards (31). At 1, 6, and 12 months, infant weight was measured using a Seca Infant Scale. Age- and sex-specific infant weight-for-age percentile was calculated following the WHO growth standards (32). Subscapular and suprailiac skinfold thicknesses (mm) were measured twice by trained study coordinators with Holtain calipers following the National Health and Nutrition Examination Survey protocols at each visit. A third measure was obtained if the difference between the first two measures was more than 1.0 mm or either of the two measures was out of reference ranges. For each anatomic site, skinfold thickness was calculated as the average value of the two closest measures. TST, a proxy for central fat mass, was calculated by taking the average of subscapular and suprailiac skinfold thickness (33, 34).

### Covariates

Maternal and infant factors previously determined to be associated with sex steroid concentrations and/or birth outcomes were selected a priori as covariates (35, 36). Maternal factors included age, race/ethnicity, education, parity, gestational age at the time of blood sample collection, early-pregnancy body mass index (BMI), smoking, and pregnancy complications (i.e., gestational hypertension and preeclampsia, and gestational diabetes). Infant factors included gestational age at birth and sex. Detail categories of covariates are described in the supplementary materials.

### Statistical Analysis

Descriptive statistics were calculated for all variables. Sex steroid hormones were log-transformed. Group-based trajectory modeling (GBTM) was used to identify growth trajectories for weight-for-age (WFA) percentile and TST measured from birth to 12 months of age (birth, 1, 6, 12 months) (37). Individuals with 2 or more measures at the study visits were included in the identification of growth trajectories. Trajectories were selected based on Bayesian information criterion (BIC). To identify potential sensitive windows when prenatal sex steroid hormones may impact fetal/infant growth, path analysis was conducted to simultaneously assess individual sex steroids at all trimesters and their independent associations with birth and infant growth outcomes using structural equation modeling adjusting for covariates (Supplementary Fig. 2). Bias-corrected bootstrap methods were used to estimate the confidence interval for the direct effect of individual sex steroids in each trimester in the path analysis. Additionally, due to prior evidence indicating sex differences in fetal growth, we examined the modification effects of infant sex by including interactions between infant sex and individual sex steroid concentrations in each trimester using linear regression models for birth outcomes and multinomial logistic regression models for infant growth trajectories. To understand the effect of birth outcomes on the relationship between prenatal sex steroids and postnatal growth, we fitted the multinomial logistic regression models with and without birth size as a covariate. All analyses were conducted using STATA 18.0 (StataCorp, College Station, TX, USA).

## Results

### Characteristics of the Study Cohort

**Figure 1:**
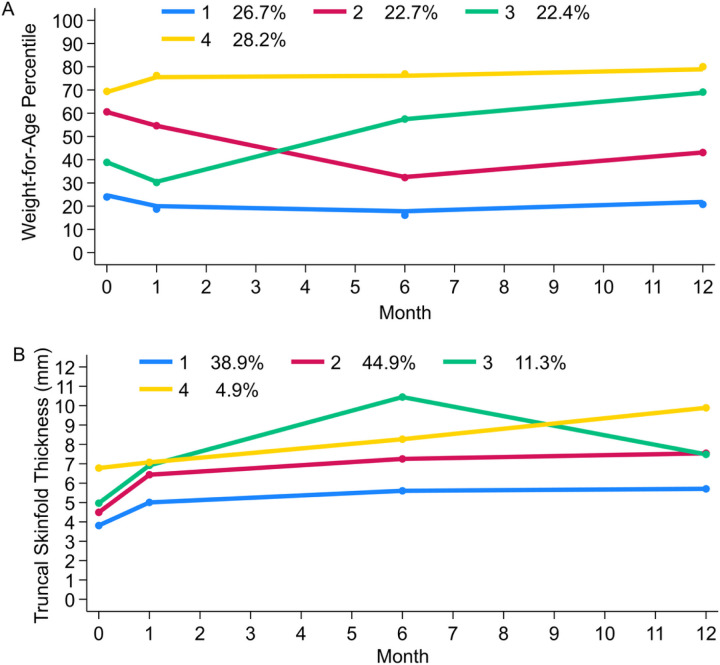
Growth trajectory patterns during infancy. A. Weight-for-age percentile growth patterns. B. Truncal skinfold thickness growth patterns.

The majority of the mothers were Caucasian (62.7%), overweight/obesity in early pregnancy (63.6%), and had education beyond high school (67.5%) (Table 1). Maternal serum estrogen levels (p < 0.001), but not testosterone levels (TT: p = 0.48; fT: p = 0.09), increased during pregnancy. Four growth trajectories were identified for WFA percentiles: (1) low stable WFA percentiles (W1: 26.7%); (2) declining WFA percentiles in early infancy (W2: 22.7%); (3) accelerating WFA percentiles (W3: 22.4%); and (4) high stable WFA percentiles (W4: 28.2%) (Fig. 1A). Four growth trajectories were also identified for TST: (1) low stable TST (T1: 38.9%); (2) moderate stable TST (T2: 44.9%); (3) accelerating TST through 6 months followed by decelerating TST (T3: 11.3%); and (4) high TST that steadily increased through 12 months of age (T4: 4.9%) (Fig. 1B). The correlation between WFA and TST trajectories was relatively weak (Spearman’s ρ = 0.29, p < 0.01)

### Associations of Prenatal Sex Steroid Hormones with Birth Size

**Figure 2:**
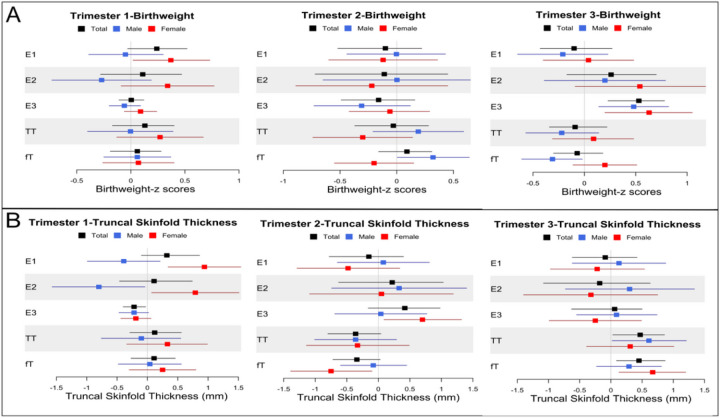
Sex differences in the associations between sex steroid hormones and birth outcomes. A. The associations between sex steroid hormones and birthweight z scores by trimester. B. The associations between sex steroid hormones and truncal skinfold thickness at birth by trimester. Black: total participants; Blue: male infants; Red: female infants.

Birthweight z-scores. Direct associations between individual sex steroid hormones in each trimester and birth outcomes were estimated using path analysis (Table 2). E3 levels in the 3rd trimester were directly associated with higher birthweight z-scores in the full cohort (β = 0.53, 95% CI: 0.23, 0.78) and in both sexes. Sex differences were evident in the associations between other sex steroid hormones and birthweight (Fig. 2A and Table 2). In female infants, higher E1 concentrations in the 1st trimester were associated with greater birthweight z-scores (E1: β = 0.37, 95% CI: 0.02, 0.73) whereas associations in males were non-significant. In male infants, fT levels in the 2nd trimester were positively associated with birthweight z-scores (β = 0.32, 95% CI: 0.005, 0.64), whereas the association was reversed in the 3rd trimester (β= −0.31, 95% CI: −0.61, −0.02). Associations between TT and birthweight z-scores were similar, but weaker and non-significant (Table 2).

TST at birth. TST was considered a proxy for central adiposity. The patterns of associations for TST at birth were relatively different from those for birthweight. E3 in the 1st trimester was inversely associated with TST (mm) at birth (β= −0.22, 95% CI: −0.40, −0.03), a trend that was consistent in male and female infants. Again, sex differences were evident in the associations between other estrogens and TST at birth (Fig. 2B and Table 2). In the 1st trimester, higher E1 and E2 levels were associated with greater TST in female infants (E1: β = 0.94, 95% CI: 0.34, 1.54; E2: β = 0.79, 95% CI: 0.07, 1.51), whereas in male infants, 1st trimester E2 was associated with lower TST (β= −0.80, 95% CI: −1.57, −0.04). In the 2nd trimester, E3 was positively associated with TST in female (β = 0.70, 95% CI: 0.09, 1.32), but not male, infants. Additionally, in the full cohort TT and fT in the 2nd trimester showed a trend towards inverse associations with TST at birth (TT: β= −0.36, 95% CI: −0.80, 0.04; fT: β= −0.34, 95% CI: −0.72, 0.03), but these associations were reversed in the 3rd trimester (TT: β = 0.47, 95% CI: 0.03, 0.86; fT: β = 0.45, 95% CI: 0.09, 0.87). Associations between testosterone and TST at birth tended to be similar among male and female infants.

### Associations of Prenatal Sex Steroid Hormones with Infant Growth Trajectories

Infant WFA trajectories. Using the low stable WFA trajectory (W1) as the referent, 3rd trimester E3 was associated with higher probability (β = 2.10, 95% CI: 0.18, 3.27) of membership in the declining WFA trajectory (W2, Table 3). This association was stronger in male infants (Supplementary Table 1). Numerous sex differences were evident in the associations between other sex steroid hormones and WFA trajectories (Supplementary Table 1). In male infants, 1st trimester E1 and E2 were associated with lower probability of the declining WFA trajectory (W2: E1: β= −2.18, 95% CI: −3.85, −0.50; E2: β= −2.57, 95% CI: −4.78, −0.36) and the high stable WFA trajectory (W4: E1: β= −1.50, 95% CI: −2.99, −0.01; E2: β= −2.30, 95% CI: −4.32, −0.29). These associations tended to be reversed and non-significant in female infants. Similarly, the associations of testosterone with WFA trajectories were in opposite directions in male and female infants. In male infants, 2nd trimester TT and fT were associated with higher probability of the declining WFA and high stable WFA trajectories (W2 and W4), while 3rd trimester TT and fT were associated with lower probability of the accelerating WFA and high stable WFA trajectories (W3 and W4). These associations were again reversed in female infants. Finally, 1st trimester TT was associated with higher probability of the high stable WFA trajectory (W4) in female infants (β = 2.17, 95% CI: 0.26, 4.09).

Infant TST trajectories. Few associations between prenatal sex steroid hormones and infant TST trajectories were observed. Using the low stable TST trajectory (T1) as the referent, 2nd trimester E3 was associated with higher probability of the moderate stable TST trajectory (T2) (β = 1.67, 95% CI: 0.33, 2.82) (Table 3). This association was consistent in both male and female infants (Supplementary Table 1). Sex differences were not evident in the associations between sex steroid hormones and TST trajectories (Supplementary Table 1). Of note, the comparisons of sex steroid hormones between the high accelerating TST trajectory (T4) and the low stable TST trajectory (T1) were not estimated due to limited number of infants with the high accelerating TST trajectory (T4).

### Associations of Prenatal Sex Steroid Hormones with Infant Growth Trajectories Adjusting for Birth Size

To evaluate the associations between prenatal sex steroid hormones and postnatal growth beyond birthweight and adiposity at birth, we refitted models with additional adjustment for birth anthropometrical measures. Birthweight was associated with the WFA trajectories (p < 0.001), and TST at birth was also associated with the TST trajectories (p ≤ 0.001). Overall, when including birthweight, models had the same directions of associations between sex steroid hormones and WFA trajectories, however the magnitude of associations tended to be enhanced in male infants and attenuated magnitude in female infants compared to models without adjustment of birthweight (Table 4). Particularly, in male infants, the associations of 1st trimester TT and fT with lower probability of the declining WFA trajectory (W2; TT: β= −3.64, 95% CI: −6.70, −0.58; fT: β= −2.56, 95% CI: −4.96, −0.16) and the high stable WFA trajectory (W4: TT: β= −3.53, 95% CI: −6.63, −0.43; fT: β= −2.47, 95% CI: −4.85, −0.10) were strengthened. Also, 2nd trimester TT was associated with increased probability of the accelerating WFA trajectory (W3; β = 2.89, 95% CI: 0.03, 5.76). In female infants the associations of TT and fT with the high stable WFA trajectory (W4) were attenuated when birthweight was included in the model.

When including TST at birth, the associations of 2nd trimester E3 with the moderate stable TST trajectory (T2) were attenuated in both male and female infants. But, in female infants, 1st trimester TT was associated with lower probability of the accelerating and decelerating TST trajectory (T3; β= −3.76, 95% CI: −6.91, −0.61), while the relationship was reversed in the 2nd trimester (β = 4.88, 95% CI: 0.54, 9.23).

## Discussion

To date, this study is the most comprehensive evaluation of the associations between prenatal maternal sex steroid hormones and fetal/infant growth with 4 major sex steroid hormones measured in all trimesters and 4 timepoints of infant anthropometric assessments. Our findings reveal the complex interplay between prenatal sex steroid hormones, birth size, and postnatal growth, with implications for understanding the developmental origins of childhood obesity. Notably, the associations between prenatal sex steroid hormones and both birth size and postnatal growth were found to vary by the specific individual sex steroids and the timing of pregnancy. Furthermore, the study uncovered significant sex differences in these associations, suggesting potential underlying mechanisms that may differ between male and female infants. Importantly, the observed associations between prenatal sex steroid hormones and postnatal weight and adiposity growth were beyond birth size, underscoring the unique role of prenatal sex steroids in shaping later childhood growth.

Specifically for birth size, our finding of the positive association between 3rd trimester E3 and birthweight aligned with prior findings (16, 17, 19–21). However, limited research has explored the associations of other sex steroid hormones with birthweight, as well as their interactions with infant sex. To our knowledge, the associations between prenatal sex steroid hormones and neonatal adiposity have not been investigated, although neonatal adiposity has been linked to later childhood obesity (3). This study found that 1st trimester E1 and E2 were associated with increased birthweight and neonatal TST in female infants after adjustment of early-pregnancy BMI. Two prior studies assessing 1st trimester E2 reported inconsistent results, with one only assessing female infants and showing a similar but non-significant trend of birthweight (21), and the other finding no associations with birthweight without assessing sex differences (24). Hence, early-pregnancy E1/E2 is potentially associated with fetal growth in female infants, which warrants further investigation.

Additionally, we observed that fT, the bioactive form of TT, in the 2nd trimester were positively associated with birthweight in male infants, while the associations were reversed in the 3rd trimester. TT showed similar trends as fT. The association of 3rd trimester, but not 2nd trimester, testosterone with birthweight was consistent with previous findings (23, 25, 38). But a prior study found higher 2nd trimester TT was associated with lower birthweights in infants of both sexes (23). Contrary to the associations with birthweight, 3rd trimester TT and fT were positively associated with neonatal TST in both sexes in this study. These findings underscored the need for further investigation into the specific relationships between prenatal sex steroid hormones and birth size, while considering sex-specific associations and the timing of hormone exposure.

For weight growth trajectories during infancy, unlike the limited previous research, which predominantly focused on 3rd trimester sex steroid hormones (16, 25, 26), this study assessed multiple sex steroid hormones across pregnancy. Additionally, the associations with infant adiposity growth trajectories were assessed in this study, as infant adiposity has been associated with childhood adiposity and cardiovascular risks (39). Specifically, a negative association between 3rd trimester E3 and the declining WFA trajectory observed in this study contrasted with a prior study that found a correlation between E3 and children’s weight at two years of age (16). This difference in findings might be attributed to the prior study’s focus on weight rather than weight trajectories and its lack of adjustment for birthweight. Additionally, this study found that associations between 1st trimester E1 and E2 and WFA growth trajectories were stronger in male infants. Also, dynamic associations were observed between E1 in the 2nd and 3rd trimesters with postnatal TST changes in infants of both sexes.

With regards to testosterone, sex differences were evident in the associations between TT/fT and postnatal growth independent of birth size. Particularly, 1st and 3rd trimester TT/fT tended to be associated with a lower probability of the declining (W2), accelerating (W3), and high stable (W4) WFA trajectories compared to the low stable WFA trajectory (W1) in male infants, while the associations were reversed in relation to 2nd trimester TT/fT. Two prior studies primarily focused on 3rd trimester TT and androgen activity, with one study in a small sample (n = 49) reporting a positive association with rapid weight gain (birth to 6 months) in male infants (25), and the other identifying an association with a higher probability of an accelerated catch-up growth pattern compared to a consistent high weight pattern in male children (26). We observed a similar trend of 3rd trimester TT with a high probability of accelerating WFA trajectory (W3) compared to high stable WFA trajectory (W4) in male infants (not shown). Interestingly, the associations between prenatal testosterone and postnatal TST trajectories were more noticeable in female infants. Overall, these findings emphasized the importance of considering longitudinal growth patterns, sexspecific differences, and controlling for birth size when assessing the impact of prenatal hormone exposure on postnatal growth with potential implications for understanding the developmental origins of childhood obesity.

The findings of this study underscored the multifaceted mechanisms potentially involved in the role of prenatal hormone exposure in fetal and postnatal growth. During early pregnancy, E1 and E2, the primary estrogens (40), stimulate angiogenesis, vascularization, blood flow in myometrial and placental arteries, and facilitating nutrient transportation from the placenta to the growing fetus through regulating uteroplacental expression of glucose transporters.(9, 10). During mid-late pregnancy, E3 is mainly synthesized by the placenta from dehydroepiandrosterone sulfate produced by fetal adrenal glands after 10 weeks of gestation and becomes the principle estrogen (16, 40). Therefore, E3 is an indicator of the function of placental sulfatases and fetal adrenal glands, which has been postulated to reflect fetal well-being (16, 41). E3 has also been proposed to regulate uteroplacental vascularization and blood flow in late pregnancy, but its full physiological function remains to be elucidated (42). Regarding postnatal growth, estrogens have been found to interact with several epigenetic enzymes and regulate epigenetic modifications (12, 43). Particularly, prenatal E3 exposure affects epigenetic modification in brain, muscle, and adipose tissues that may be involved in postnatal adiposity and growth (12).

We observed sex-specific difference in the associations between prenatal testosterone exposure and fetal and postnatal growth and adiposity. The role of 2nd trimester testosterone appeared to differ from that of testosterone in the 1st and/or 3rd trimesters. These variations in infant sex and timing of pregnancy may be attributed to the peak fetal testosterone production in late 1st and early 2nd trimesters in male fetuses (44). Testosterone of fetal origin may promote musculoskeletal development in male fetuses during this stage (45). However, testosterone of maternal origin downregulated placental amino acid transport activity in a rat model (11). Also, maternal testosterone reduced uterine artery blood flow and affected placental vasculature particularly in male rat fetuses (46). This evidence could explain how high maternal testosterone levels were associated with lower birthweight, particularly in male fetuses. Furthermore, testosterone has been implicated in promoting visceral preadipocyte proliferation and adiposity in human (13, 47), with sex-specific responses likely influenced by sex dimorphism in adipose tissue biology, such as differential expression of sex steroid receptors in adipose tissue, the number of adipocyte precursor cells, and differential programming by sex chromosome (27). Additionally, testosterone interacts with epigenetic enzymes and can regulate epigenetic modifications (48), potentially influencing postnatal growth and adiposity. Finally, postnatal endogenous testosterone production in male infants before 6 months of age may contribute to sex differences in postnatal growth (49). The association between prenatal maternal and postnatal infant testosterone levels remains unclear and warrants further investigation. If maternal hormones play a role in programming the developing infant hormone axis during pregnancy, it may offer another mechanism for understanding associations between prenatal maternal sex steroids and postnatal infant size.

The strengths of this study include: (1) the prospective repeated measures of the major estrogens and testosterone across all three trimesters; (2) sensitive LC-MS/MS and equilibrium dialysis method were used to quantify estrogens and testosterone, particularly the low concentrations of E3 in early pregnancy and testosterone across pregnancy; (3) by using path analysis models and including interactions with infant sex, we were able to estimate direct associations of sex steroids in three trimesters simultaneously, giving insight into sensitive periods of hormone exposure and sex-specific responses; (4) birth size was adjusted in the models of postnatal growth, underscoring the role of prenatal sex steroid hormones in postnatal growth beyond birth size. Several caveats are important to consider when interpreting the findings of this study. We focused on estrogens and testosterone and did not assess additional prenatal hormones, such as dihydrotestosterone, and progesterone, that may mediate the role of estrogens and testosterone in fetal and postnatal growth (26). Also, the concentrations of fT were low, making them susceptible to methodological issues. Therefore, the results of fT should be interpreted alongside those of TT. Additionally, we used a relatively crude measure of adiposity, and more advanced tools, such as dual-energy x-ray absorptiometry, magnetic resonance imaging, or air displacement plethysmography may provide more accurate measures.

## Conclusion

Prenatal sex steroids were associated with fetal and postnatal growth at different stages of pregnancy. Sex differences were evident in these relationships and require further investigation. Furthermore, the associations between prenatal sex steroids and postnatal growth might be beyond birth anthropometry. These findings contribute to a deeper understanding of the complex interplay between prenatal sex steroid hormones and fetal and postnatal growth, emphasizing the need for further research to elucidate the underlying mechanisms and potential long-term implications for childhood obesity.

## Figures and Tables

**Table 1:** Characteristics of UPSIDE mother-infant dyads in this study

Variable^a^		All Participants (n = 252)
**Mothers**		
Age (years)		29.3 ± 4.4
Race/Ethnicity		
White, Non-Hispanic		158 (62.7%)
Black, Non-Hispanic		54 (21.4%)
Hispanic		21 (8.3%)
Others		19 (7.5%)
Nulliparous		84 (33.3%)
High school or less education		82 (32.5%)
Smoking during pregnancy		17 (6.8%)
Gestational hypertension or preeclampsia		30 (11.9%)
Gestational diabetes		14 (5.6%)
Early-pregnancy BMI		
Normal/underweight		117 (46.4%)
Overweight		62 (24.6%)
Obesity		73 (29.0%)
Trimester 1	E1 (pg/mL)	1152.4 ± 900.5
	E2 (pg/mL)	1853.9 ± 1017.1
	E3 (pg/mL)	291.3 ± 260.3
	TT (ng/dL)	69.1 ± 44.6
	fT (ng/dL)	0.4 ± 0.3
Trimester 2	E1 (pg/mL)	4255.6 ± 2987.7
	E2 (pg/mL)	6539.7 ± 3021.5
	E3 (pg/mL)	3247.4 ± 1337.1
	TT (ng/dL)	77.4 ± 53.6
	fT (ng/dL)	0.4 ± 0.3
Trimester 3	E1 (pg/mL)	6664.7 ± 4492.2
	E2 (pg/mL)	11868.0 ± 4756.9
	E3 (pg/mL)	7026.4 ± 3147.2
	TT (ng/dL)	75.6 ± 55.3
	fT (ng/dL)	0.4 ± 0.3
**Infants**		
Gestational age (weeks)		39.7 ± 1.1
Female		126 (50%)
Birthweight (g)		3422 ± 472
WFA percentile at birth		48.3 ± 26.7
WFA percentile at 1 month (n = 199)		46.0 ± 28.7
WFA percentile at 6 months (n = 193)		46.4 ± 29.1
WFA percentile at 12 months (n = 168)		53.6 ± 29.4
TST (mm) at birth (n = 234)		4.4 ± 1.3
TST (mm) at 1 month (n = 202)		5.9 ± 1.5
TST (mm) at 6 months (n = 189)		7.0 ± 1.8
TST (mm) at 12 months (n = 161)		6.9 ± 1.6

Note.

a Continuous variables are summarized using mean and standard deviation; Categorical variables are summarized using count and percentage. E1, estrone; E2, estradiol; E3, estiol; TT, total testosterone; fT, free testosterone; WFA, weight for age; TST, truncal skinfold thickness.

**Table 2:** Associations between prenatal sex steroid hormones and birthweight and central adiposity in total samples and by infant sex.

		Total				Male				Female			
		Birthweight (z scores)		Truncal skinfold thickness (mm)		Birthweight (z scores)		Truncal skinfold thickness (mm)		Birthweight (z scores)		Truncal skinfold thickness (mm)	
		B	95% CI	β	95% CI	β	95% CI	β	95% CI	β	95% CI*	β	95% CI
Estrone	Trimester 1	0.24	−0.03, 0.52	0.32	−0.10, 0.86	−0.05	−0.39, 0.30	−0.39*	−0.99, 0.21	**0.37**	**0.02, 0.73**	**0.94** *	**0.34, 1.54**
	Trimester 2	−0.10	−0.52, 0.22	−0.15	−0.78, 0.40	−0.003	−0.44, 0.43	0.08	−0.65, 0.81	−0.12	−0.60, 0.36	−0.48	−1.29, 0.34
	Trimester 3	−0.10	−0.43, 0.27	−0.09	−0.62, 0.42	−0.21	−0.65, 0.23	0.13	−0.62, 0.88	0.04	−0.40, 0.48	−0.22	−0.97, 0.54
Estradiol	Trimester 1	0.11	−0.28, 0.47	0.11	−0.46, 0.74	−0.27*	−0.73, 0.19	**−0.80** *	**−1.57, −0.04**	0.34*	−0.09, 0.77	**0.79** *	**0.07, 1.51**
	Trimester 2	−0.11	−0.72, 0.45	0.22	−0.63, 1.03	0.002	−0.65, 0.65	0.33	−0.74, 1.40	−0.22	−0.89, 0.45	0.05	−1.09, 1.19
	Trimester 3	0.26	−0.17, 0.70	−0.18	−1.08, 0.63	0.20	−0.39, 0.79	0.30	−0.73, 1.34	0.54	−0.09, 1.18	−0.32	−1.40, 0.75
Estriol	Trimester 1	0.004	−0.11, 0.12	**−0.22**	**−0.40, −0.03**	−0.06	−0.20, 0.09	−0.22	−0.47, 0.02	0.09	−0.06, 0.24	−0.19	−0.44, 0.06
	Trimester 2	−0.16	−0.49, 0.16	0.42	−0.16, 0.98	−0.31	−0.73, 0.12	0.04	−0.69, 0.77	−0.06	−0.42, 0.29	**0.70**	**0.09, 1.32**
	Trimester 3	**0.53**	**0.23, 0.78**	0.06	−0.63, 0.50	**0.48**	**0.14, 0.82**	0.09	−0.55, 0.74	**0.63**	**0.20, 1.05**	−0.25	−0.99, 0.49
Total testosterone	Trimester 1	0.13	−0.17, 0.40	0.12	−0.29, 0.56	−0.003	−0.40, 0.39	−0.10	−0.76, 0.55	0.27	−0.13, 0.67	0.33	−0.34, 0.99
	Trimester 2	−0.03	−0.37, 0.28	−0.36	−0.80, 0.04	0.19	−0.21, 0.59	−0.36	−1.01, 0.29	−0.30	−0.74, 0.14	−0.33	−1.14, 0.49
	Trimester 3	−0.09	−0.34, 0.22	**0.47**	**0.03, 0.86**	−0.22	−0.57, 0.14	**0.61**	**0.02, 1.21**	0.09	−0.31, 0.48	0.31	−0.39, 1.01
Free testosterone	Trimester 1	0.06	−0.19, 0.28	0.11	−0.27, 0.46	0.06	−0.25, 0.37	0.04	−0.48, 0.56	0.07	−0.26, 0.40	0.25	−0.30, 0.80
	Trimester 2	0.09	−0.16, 0.31	−0.34	−0.72, 0.03	**0.32** *	**0.005, 0.64**	−0.08	−0.60, 0.45	−0.20*	−0.55, 0.15	**−0.75**	**−1.39, −0.10**
	Trimester 3	−0.07	−0.30, 0.18	**0.45**	**0.09, 0.87**	**−0.31** *	**−0.61, −0.02**	0.29	−0.23, 0.81	0.20*	−0.11, 0.51	**0.67**	**0.13, 1.20**

Note. The sample size for birthweight was 252 and the sample size for truncal skinfold thickness was 234. Direct associations of individual sex steroid hormones at each trimester were estimated using path analysis in the total samples and linear regression models for sex differences. Sex steroid hormones were log transformed. Model was adjusted for gestational age of blood draw, maternal age, race/ethnicity, parity, early-pregnancy BMI, gestational hypertension and preeclampsia, gestational diabetes, education, smoking during pregnancy, infant sex and gestational age.

* indicates interaction with infant sex significant at p < 0.05.

**Table 3:** The direct association of sex steroid hormone at each trimester and weight-for-age and central adiposity growth patterns during infancy.

		Weight-for-age percentile trajectories (n = 218) β, 95% CI^a^				Truncal skinfold thickness trajectories (n = 215) β, 95% CI^a^		
		W1 Low stable	W2 Declining	W3 Accelerating	W4 High stable	T1 Low stable	T2 Moderate stable	T3 Accelerating-decelerating
Estrone	Trimester 1	Ref	−0.43 (−1.87, 0.97)	0.67 (−1.04, 1.82)	−0.44 (−1.75, 0.87)	Ref	0.05 (−1.09, 0.97)	1.52 (−1.20, 6.14)
	Trimester 2		0.70 (−1.14, 2.35)	−0.73 (−2.47, 1.09)	1.10 (−0.86, 2.79)		0.96 (−0.30, 2.22)	−2.58 (−6.71, 1.20)
	Trimester 3		−0.13 (−1.77, 1.30)	0.55 (−0.94, 2.04)	−0.74 (−2.25, 1.07)		−1.16 (−2.40, 0.06)	2.19 (−4.42, 5.01)
Estradiol	Trimester 1	Ref	−0.48 (−2.33, 1.17)	−0.32 (−2.12, 1.42)	−0.83 (−2.51, 1.08)	Ref	0.13 (−1.28, 1.32)	1.15 (−2.68, 5.19)
	Trimester 2		0.59 (−2.30, 3.31)	−0.61 (−3.21, 2.26)	1.38 (−1.36, 4.51)		1.35 (−0.47, 3.41)	−1.83 (−6.23, 5.68)
	Trimester 3		1.31 (−1.23, 3.58)	1.94 (−0.97, 3.62)	0.04 (−2.71, 2.24)		−0.90 (−2.80, 0.80)	0.86 (−3.01, 4.53)
Estriol	Trimester 1	Ref	0.12 (−0.58, 0.60)	−0.12 (−0.70, 0.55)	0.03 (−0.59, 0.64)	Ref	−0.13 (−0.55, 0.28)	−0.66 (−1.75, 0.74)
	Trimester 2		0.35 (−1.29, 2.05)	−0.35 (−1.93, 1.57)	0.39 (−1.18, 2.23)		**1.67 (0.33, 2.82)**	0.40 (−1.87, 2.98)
	Trimester 3		**2.10 (0.18, 3.27)**	0.82 (−0.84, 2.12)	1.09 (−0.74, 2.40)		−0.41 (−1.50, 0.66)	−0.35 (−2.48, 3.27)
Total testosterone	Trimester 1	Ref	0.03 (−1.59, 1.53)	−0.09 (−1.99, 1.44)	0.44 (−1.20, 1.81)	Ref	−0.79 (−1.85, 0.26)	−0.90 (−3.94, 4.20)
	Trimester 2		0.56 (−0.95, 2.73)	0.40 (−1.27, 2.46)	0.04 (−1.49, 1.83)		0.13 (−1.27, 1.34)	0.15 (−3.42, 4.54)
	Trimester 3		−0.93 (−2.08, 0.54)	−0.26 (−1.62, 1.20)	−0.78 (−1.97, 0.66)		0.57 (−0.43, 1.68)	0.28 (−2.80, 2.31)
Free testosterone	Trimester 1	Ref	−0.25 (−1.47, 0.82)	−0.08 (−1.45, 1.30)	−0.01 (−1.29, 1.15)	Ref	−0.46 (−1.35, 0.37)	0.07 (−3.42, 2.38)
	Trimester 2		0.34 (−0.78, 1.64)	0.35 (−1.02, 1.58)	0.11 (−0.94, 1.32)		−0.20 (−1.07, 0.67)	−0.02 (−2.31, 3.52)
	Trimester 3		−0.42 (−1.50, 0.85)	−0.21 (−1.21, 1.02)	−0.24 (−1.30, 0.87)		0.31 (−0.47, 1.20)	−0.47(−2.59, 2.08)

Note. Path analysis was used to estimate direct association. Sex steroid hormones were log transformed. Model was adjusted for gestational age of blood draw, maternal age, race/ethnicity, parity, early-pregnancy BMI, gestational hypertension and preeclampsia, gestational diabetes, education, smoking during pregnancy, infant sex and gestational age.

a bias-corrected bootstrap confidence interval. The associations between sex steroid hormones and T4 for truncal skinfold thickness were not estimated due to limited sample size.

**Table 4:** The direct association of sex steroid hormone at each trimester and weight-for-age and central adiposity growth patterns by infant sex adjusting for birth outcomes.

		Weight-for-age percentile trajectories (n = 218) β, 95% CI^a^				Truncal skinfold thickness trajectories (n = 215) β, 95% CI^a^		
		W1 Low stable	W2 Declining	W3 Accelerating	W4 High stable	T1 Low stable	T2 Moderate stable	T3 Accelerating-decelerating
**Male**								
Estrone	Trimester 1	Ref	−4.00* **(−6.82, −1.17)**	0.26 (−1.39, 1.91)	**−3.87 (−6.59, −1.16)**	Ref	−0.01 (−1.16, 1.14)	0.70 (−1.70, 3.09)
	Trimester 2		1.79 (−1.82, 5.40)	−0.92 (−3.05, 1.20)	1.87 (−1.70, 5.43)		0.70 (−0.81, 2.22)	−2.53 (−5.94, 0.89)
	Trimester 3		1.20 (−2.42, 4.82)	1.05 (−1.02, 3.11)	−0.09 (−3.71, 3.54)		−1.32 (−2.80, 0.15)	2.32 (−0.51, 5.15)
Estradiol	Trimester 1	Ref	−3.23* **(−6.35, −0.10)**	−0.28 (−2.24, 1.69)	−4.36* **(−7.62, −1.11)**	Ref	0.20 (−1.33, 1.74)	1.27 (−1.72, 4.25)
	Trimester 2		0.43 (−4.39, 5.25)	−1.97 (−5.32, 1.39)	1.42 (−3.52, 6.36)		1.13 (−1.12, 3.38)	−3.64 (−8.40, 1.13)
	Trimester 3		4.03 (−0.40, 8.45)	2.72 (−0.36, 5.80)	0.98 (−3.57, 5.53)		−1.25 (−3.31, 0.82)	2.44 (−1.97, 6.85)
Estriol	Trimester 1	Ref	−0.45 (−1.44, 0.54)	−0.17 (−0.85, 0.51)	−0.88* (−1.89, 0.14)	Ref	−0.17 (−0.65, 0.30)	−0.29 (−1.31, 0.72)
	Trimester 2		0.58 (−2.63, 3.78)	−1.18 (−3.36, 0.99)	2.23 (−1.03, 5.48)		1.42 (−0.15, 2.99)	−1.21 (−4.10, 1.68)
	Trimester 3		3.93* **(0.99, 6.88)**	1.03 (−0.72, 2.78)	1.22 (−1.47, 3.91)		−0.53 (−1.87, 0.82)	−1.32 (−4.11, 1.47)
Total testosterone	Trimester 1	Ref	−3.64* **(−6.70, −0.58)**	−0.51 (−2.68, 1.65)	−3.53* **(−6.63, −0.43)**	Ref	−0.65 (−2.01, 0.71)	−0.38 (−2.89, 2.14)
	Trimester 2		4.48* **(0.55, 8.41)**	**2.89 (0.03, 5.76)**	3.72* (−0.22, 7.66)		0.55 (−1.05, 2.14)	−1.76* (−5.49, 1.97)
	Trimester 3		−2.80 (−5.80, 0.19)	−2.69* **(−4.69, −0.69)**	−3.67* **(−6.66, −0.69)**		−0.06 (−1.35, 1.24)	0.50 (−2.08, 3.07)
Free testosterone	Trimester 1	Ref	**−2.56 (−4.96, −0.16)**	0.16 (−1.41, 1.73)	**−2.47 (−4.85, −0.10)**	Ref	−0.51 (−1.57, 0.56)	−0.06 (−1.83, 1.72)
	Trimester 2		1.68 (−0.96, 4.31)	1.62 (−0.45, 3.68)	1.86* (−0.82, 4.54)		0.06 (−1.11, 1.24)	−0.71 (−3.13, 1.70)
	Trimester 3		−1.21 (−3.35, 0.93)	−1.72* **(−3.32, −0.12)**	−2.79* **(−5.02, −0.56)**		0.19 (−0.87, 1.25)	−0.34 (−2.81, 2.13)
**Female**								
Estrone	Trimester 1	Ref	0.93* (−1.34, 3.20)	1.24 (−0.74, 3.22)	−0.75 (−3.26, 1.75)	Ref	−0.04 (−1.31, 1.23)	2.55 (−0.64, 5.74)
	Trimester 2		0.77 (−1.58, 3.12)	−0.003 (−2.12, 2.11)	2.53 (−0.11, 5.16)		1.03 (−0.59, 2.65)	−3.12 (−7.61, 1.36)
	Trimester 3		−1.84 (−4.46, 0.78)	−0.73 (−2.95, 1.49)	−2.15 (−5.12, 0.81)		−0.80 (−2.39, 0.79)	3.67 (−0.58, 7.92)
Estradiol	Trimester 1	Ref	1.75* (−0.86, 4.37)	−1.01 (−3.38, 1.36)	−0.23* (−3.00, 2.55)	Ref	0.85 (−0.67, 2.36)	1.54 (−1.72, 4.79)
	Trimester 2		−1.22 (−4.79, 2.35)	1.33 (−1.75, 4.41)	1.79 (−2.19, 5.77)		1.17 (−1.14, 3.49)	−2.18 (−7.05, 2.69)
	Trimester 3		−0.39 (−3.95, 3.18)	1.92 (−1.25, 5.08)	−2.15 (−6.05, 1.75)		−1.67 (−3.91, 0.56)	1.46 (−2.64, 5.56)
Estriol	Trimester 1	Ref	0.70 (−0.17, 1.58)	−0.03 (−0.76, 0.69)	0.77* (−0.18, 1.72)	Ref	0.12 (−0.39, 0.62)	−0.50 (−1.45, 0.46)
	Trimester 2		1.64 (−0.33, 3.61)	0.47 (−1.10, 2.03)	1.55 (−0.61, 3.72)		1.22 (−0.20, 2.64)	1.29 (−1.61, 4.19)
	Trimester 3		−0.11* (−2.53, 2.31)	0.49 (−1.57, 2.54)	−0.89 (−3.53, 1.74)		−0.40 (−1.94, 1.14)	0.58 (−2.32, 3.48)
Total testosterone	Trimester 1	Ref	0.50* (−1.60, 2.61)	−0.74 (−2.63, 1.15)	1.53* (−1.12, 4.19)	Ref	−1.10 (−2.48, 0.29)	**−3.76 (−6.91, −0.61)**
	Trimester 2		−1.80* (−4.45, 0.84)	−0.96 (−3.59, 1.66)	−3.11* (−6.53, 0.32)		0.62 (−1.15, 2.39)	4.88* **(0.54, 9.23)**
	Trimester 3		0.12 (−1.87, 2.11)	1.09* (−0.97, 3.16)	1.54* (−1.03, 4.10)		−0.05 (−1.43, 1.34)	−0.47 (−3.46, 2.53)
Free testosterone	Trimester 1	Ref	−0.64 (−2.34, 1.05)	−1.15 (−2.76, 0.46)	−0.05 (−1.99, 1.88)	Ref	−0.52 (−1.69, 0.65)	−0.91 (−3.51, 1.70)
	Trimester 2		−1.29 (−3.27, 0.68)	−0.34 (−2.08, 1.41)	−2.08* (−4.48, 0.31)		−0.11 (−1.45, 1.22)	2.26 (−0.79, 5.32)
	Trimester 3		0.22 (−1.51, 1.96)	0.70* (−0.98, 2.39)	1.39* (−0.63, 3.41)		−0.42 (−1.51, 0.67)	−1.27 (−3.40, 0.85)

Note. Path analysis was used to estimate direct association. Sex steroid hormones were log transformed. Model was adjusted for gestational age of blood draw, maternal age, race/ethnicity, parity, early-pregnancy BMI, gestational hypertension and preeclampsia, gestational diabetes, education, smoking during pregnancy, infant sex and gestational age.

a bias-corrected bootstrap confidence interval. The associations between sex steroid hormones and T4 for truncal skinfold thickness were not estimated due to limited sample size.

* indicates interaction with infant sex significant at p < 0.05. The comparisons of sex steroid hormones between TST T4 and T1 patterns were not estimated due to limited number of infants with T4 pattern.
